# FBW7 regulates DNA interstrand cross-link repair by modulating FAAP20 degradation

**DOI:** 10.18632/oncotarget.9595

**Published:** 2016-05-25

**Authors:** Jingming Wang, Ukhyun Jo, So Young Joo, Hyungjin Kim

**Affiliations:** ^1^ Department of Pharmacological Sciences, Stony Brook University, Stony Brook, New York, USA

**Keywords:** Fanconi anemia, ubiquitin-proteasome system, FBW7, genome instability, tumor suppressor, Chromosome Section

## Abstract

Mutations that deregulate protein degradation lead to human malignancies. The SCF ubiquitin E3 ligase complex degrades key oncogenic regulators, thereby limiting their oncogenic potential. FBW7 is a substrate recognition subunit of SCF^FBW7^ and is among the most commonly mutated ubiquitin-proteasome system proteins in cancer. *FBW7*-mutated cancer cells display increased genome instability, but the molecular mechanism by which FBW7 preserves genome integrity remains elusive. Here, we demonstrate that SCF^FBW7^ regulates the stability of FAAP20, a critical component of the Fanconi anemia (FA) DNA interstrand cross-link (ICL) repair pathway. Phosphorylation of the FAAP20 degron motif by GSK3β provides a platform for recognition and polyubiquitination of FAAP20 by FBW7, and its subsequent degradation by the proteasome. Accordingly, enhanced GSK3β-FBW7 signaling disrupts the FA pathway. In cells expressing non-phosphorylatable FAAP20 mutant, the turnover of its binding partner, FANCA, is deregulated in the chromatin during DNA ICL repair, and the FA pathway is compromised. We propose that FAAP20 degradation, which is prompted by its phosphorylation, controls the dynamics of the FA core complex required for completing DNA ICL repair. Together, this study provides insights into how FBW7-mediated proteolysis regulates genome stability and how its deregulation is associated with tumorigenesis.

## INTRODUCTION

The ubiquitin-proteasome system (UPS) regulates a broad range of cellular processes by governing the cellular levels of key regulatory proteins [1]. Covalent attachment of poly-ubiquitin (Ub) to substrates by an enzymatic cascade of E1 activating, E2 conjugating, and E3 ligase activity leads to proteasome-mediated substrate destruction, thereby ensuring protein homeostasis [2]. Consequently, mutations that deregulate protein degradation are associated with many human diseases, especially cancer [3]. Disrupting balanced levels of oncoproteins or tumor suppressors by either loss of Ub E3 ligase or enhanced deubiquitinating enzyme (DUB) activity provides cancer cells with a survival advantage. Therefore, strategies that alter the tumor-specific activity of UPS enzymes have emerged as promising anti-cancer therapies [4].

Ubiquitin E3 ligases confer substrate specificity and therefore account for the existence of several hundred types of E3 ligases in the human genome [5]. Most E3 ligases function as a complex, utilizing distinct modules for substrate binding and catalytic activity. FBW7 (F-box and WD repeat domain-containing 7, also called cell division cycle mutant 4, Cdc4, in budding yeast) is a substrate recognition unit of the SCF (SKP1-CUL1-F-box protein) ubiquitin E3 ligase complex, SCF^FBW7^, a member of Cullin-RING finger domain-containing E3 ligase [6]. FBW7 directly interacts with SKP1 through its N-terminal F-box, whereas the C-terminal stretch of eight WD40 repeats makes contact with its substrates [7]. The WD40 repeats constitute an eight-bladed barrel-shaped β-propeller, forming a pocket that accommodates a conserved motif of substrates [8, 9]. Importantly, FBW7 recognizes its substrates when they are phosphorylated within the so-called Cdc4 phospho-degron (CPD) motif, which consists of the amino acid sequence L-X-pS/pT(0)-P-X-X-pS/pT(+4) [10–13]. Phosphorylation of the CPD motif determines the context of substrate degradation by SCF^FBW7^; in many cases, glycogen synthase kinase 3β (GSK3β) is responsible for the phosphorylation. By phosphorylating the central S/T residues upon recognizing priming phosphorylation at +4 (or glutamate), GSK3β generates an optimal consensus motif in a substrate, which is necessary for FBW7 binding [14].

The function of FBW7 is closely associated with tumorigenesis as SCF^FBW7^ degrades many key regulators of cell proliferation, growth, and death, including Cyclin E, *c*-Myc, Notch, and MCL-1 [15]. Accordingly, *FBW7* is one of the most frequently mutated genes in many human cancers including T cell acute lymphoblastic leukemia (T-ALL), colorectal carcinoma, and cholangiocarcinoma, to name a few, highlighting its role as a tumor suppressor [16, 17]. Genetic studies of murine *Fbw7* have also supported the tumor suppressive function of Fbw7 in a haplo-insufficient manner [18–20]. Notably, arginine residues in the WD40 domain including R465, R479, and R505, which are required for the phosphate interaction, are frequently mutated in cancer [21]. These mutations indicate that selection pressure allows the oncogenic substrates of FBW7 to evade destruction during tumorigenesis.

In addition to deregulating cell cycle and proliferation, loss of FBW7 function leads to genome instability [6, 22]. For instance, genetic disruption of the *FBW7* gene in colorectal cancer cells results in gross chromosome aberrations that are associated with micronuclei formation and spindle dysfunction [23]. Although alterations in the cell cycle due to elevated Cyclin E levels have been implicated in increased genome instability in *FBW7* mutation-associated cancers, the mechanism by which FBW7 is linked to DNA metabolism is not well established. FBW7 loss may contribute to tumorigenesis by affecting the capacity of DNA repair required for maintaining genome integrity. However, whether FBW7 directly regulates the activity of DNA repair proteins remains elusive.

Genome instability caused by a defective DNA repair system is a key hallmark of cancer [24]. The Fanconi anemia (FA) pathway is a DNA repair mechanism that resolves DNA interstrand cross-links (ICLs) encountered during DNA replication [25]. Unresolved DNA ICLs block DNA replication and transcription, leading to chromosome breakage and the formation of quadrilateral chromosomes, a source of genome instability and cellular toxicity [26]. The FA pathway also counteracts replication stress by preserving replication forks, and it is required for neutralizing the genotoxicity induced by endogenous reactive aldehydes [27, 28]. Germ-line mutations in genes that cooperate in the FA pathway causes not only FA, an inherited blood disorder, but also a predisposition to multiple cancers, highlighting the role of the FA pathway in functioning as a tumor suppressive mechanism that preserves genome integrity [29].

At least 19 gene products participate in resolving DNA ICLs, and the key step in the FA pathway is monoubiquitination of FANCD2, which recruits structure-specific nucleases to the sites of DNA damage and initiates downstream DNA repair steps, including nucleolytic incision of ICL, lesion bypass, and homologous recombination (HR) [30, 31]. FANCD2 monoubiquitination is mediated by the multi-subunit ubiquitin E3 ligase, the FA core complex, which consists of eight FANC proteins, along with several accessory proteins [32]. The FANCA subunit functions as a scaffold of the complex and is most significantly mutated among FA patients [33, 34]. Given the crucial role of FANCD2 activation in the FA pathway, the activity of the FA core complex needs to be tightly controlled by combinatorial posttranslational modifications, including phosphorylation, ubiquitination, and SUMOylation, as well as interactions among FANC subunits [35]. Our group and others have identified FAAP20 (Fanconi Anemia-Associated Protein, 20 kDa) as a new subunit of the FA core complex and shown that FAAP20 maintains the stability of FANCA through its direct interaction [36–38]. Loss of the FAAP20 interaction with FANCA impairs the integrity of the FA core complex, rendering cells hypersensitive to ICL-inducing agents. We also defined the mechanism by which FAAP20 prevents FANCA from undergoing uncontrolled degradation, which is mediated by integrated ubiquitin-SUMO signaling [39]. However, the mechanism by which FANCA-FAAP20 interaction dynamics are regulated during the course of DNA ICL repair and how its deregulation impacts the FA pathway remains poorly understood.

Here, we identify SCF^FBW7^ as a ubiquitin E3 ligase that regulates the cellular FAAP20 levels and FA pathway. Deregulation of the GSK3β- and FBW7-dependent FAAP20 degradation leads to a defect in the FA pathway, establishing a direct link between FBW7 and DNA repair. Together, this study contributes to our understanding of the role of UPS in regulating DNA repair and provides molecular insights into how the FA pathway is connected to the genome instability of FBW7-associated cancer.

## RESULTS

### The phospho-degron motif of FAAP20 is required for FAAP20 degradation

**Figure 1:**
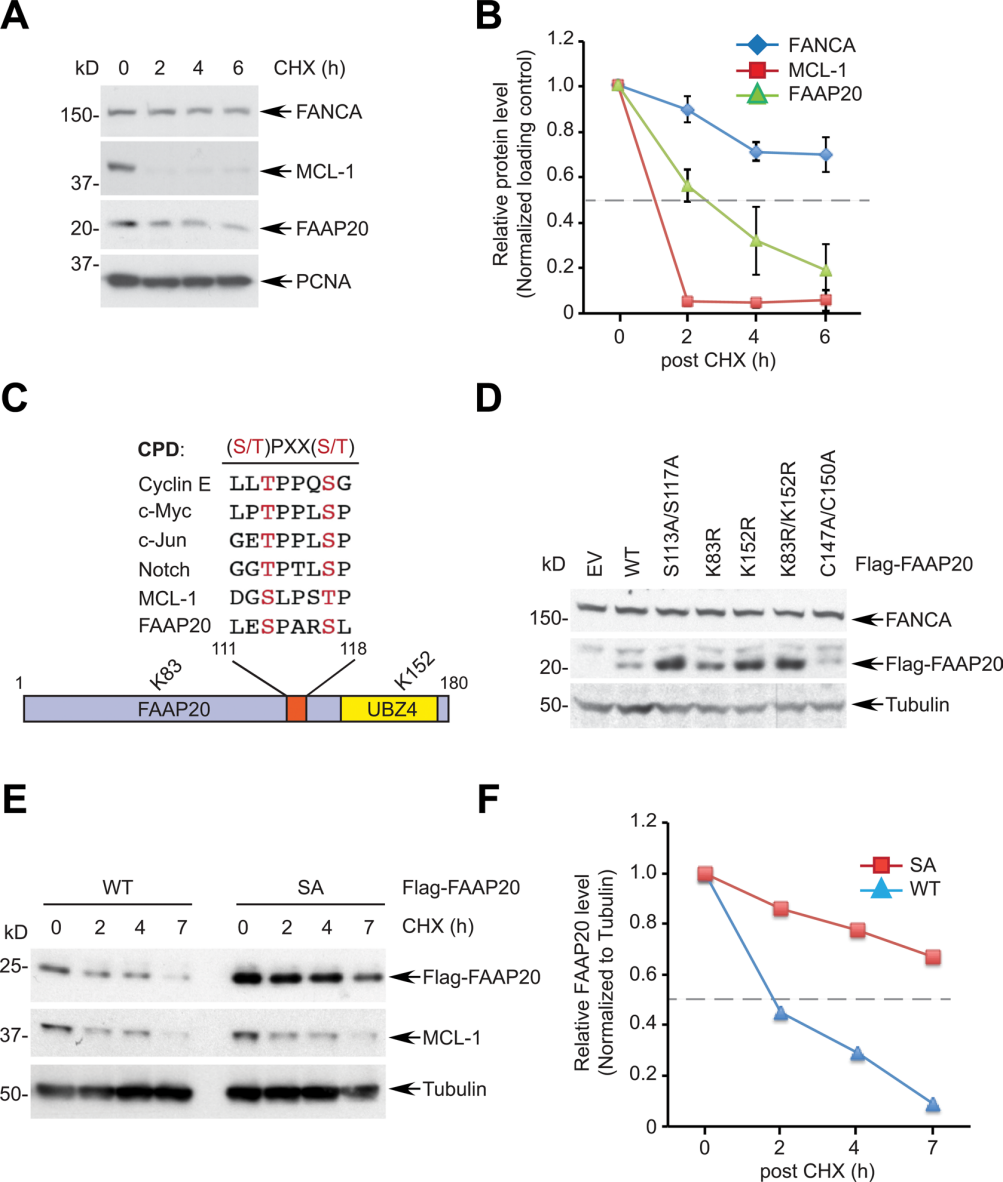
The CPD motif of FAAP20 regulates FAAP20 degradation **A.** FAAP20 is the protein with a short half-life. HeLa cells were treated with 50 μg/mL of cycloheximide (CHX) for the indicated times and cell lysates analyzed for Western blotting. **B.** Densitometry of immunoblots in **A.** acquired by ImageJ. The dotted line denotes half-life. Error bars indicate SD from two independent experiments. **C.** Alignment of the FAAP20 CPD motif with known FBW7 substrates. A schematic of the FAAP20 protein is shown below. **D.** Mutation either in the CPD motif or in two lysine residues of FAAP20 increases the cellular FAAP20 levels. HeLa cells transfected with the plasmids encoding FAAP20 variants were analyzed by Western blotting. EV: empty vector, C147A/C150A: ubiquitin-binding zinc finger (UBZ) loss-of-function mutant **E.** Mutation in the CPD motif prolongs the half-life of FAAP20. Flag-tagged wild-type (WT) or S113A/S117A (SA) mutant were transiently expressed in HeLa cells, treated with 50 μg/mL of cycloheximide (CHX) for the indicated times, and cell lysates were analyzed by Western blotting. **F.** Densitometry of immunoblots in **E.** acquired by ImageJ.

As the FANCA-FAAP20 interaction is essential for maintaining the functional FA core complex, we sought to determine how the interaction dynamics are regulated, which could dictate the efficiency of DNA ICL repair. Measuring the half-life of FANCA and FAAP20 by the cycloheximide (CHX) blocking assay showed that FAAP20 is rapidly degraded compared with FANCA, which exhibits a longer half-life, indicating that FAAP20 turnover should be tightly regulated to maintain FANCA stability (Figure 1A, 1B). Analysis of the primary amino acid sequence of FAAP20 revealed a conserved CPD motif with two phosphorylation sites at Ser113 and Ser117, suggesting that FAAP20 may be a new substrate of SCF^FBW7^ (Figure 1C). FAAP20 also contains two lysine residues at amino acids 83 and 152 that can be utilized for the polyubiquitination required for FAAP20 degradation (Figure 1C). Therefore, we expressed exogenous FAAP20 variants in HeLa cells by transfecting low levels of plasmids to determine the role of these residues in regulating the cellular FAAP20 levels. While both FAAP20 K83R and K152R single mutants had increased steady levels of FAAP20, the K152 mutation was sufficient to elevate the FAAP20 levels comparable with the K83R/K152R mutant, suggesting that K152 is a primary site for polyubiquitination (Figure 1D). As expected, the half-life of the K83R/K152R mutant was substantially increased (Figure S1A). The FAAP20 mutant that had two mutated phosphorylation sites at the CPD motif (S113A/S117A) also showed increased FAAP20 levels comparable to the K83R/K152R mutant, indicating that the intact CPD motif is required for maintaining proper cellular FAAP20 levels (Figure 1D). Indeed, the half-life of the FAAP20 SA mutant was prolonged compared with that of the wild-type, suggesting that the CPD motif is required for FAAP20 degradation (Figure 1E, 1F). Taken together, these data indicate that FAAP20 proteolysis is regulated by the intact CPD motif.

### FBW7 regulates the FAAP20 stability

**Figure 2:**
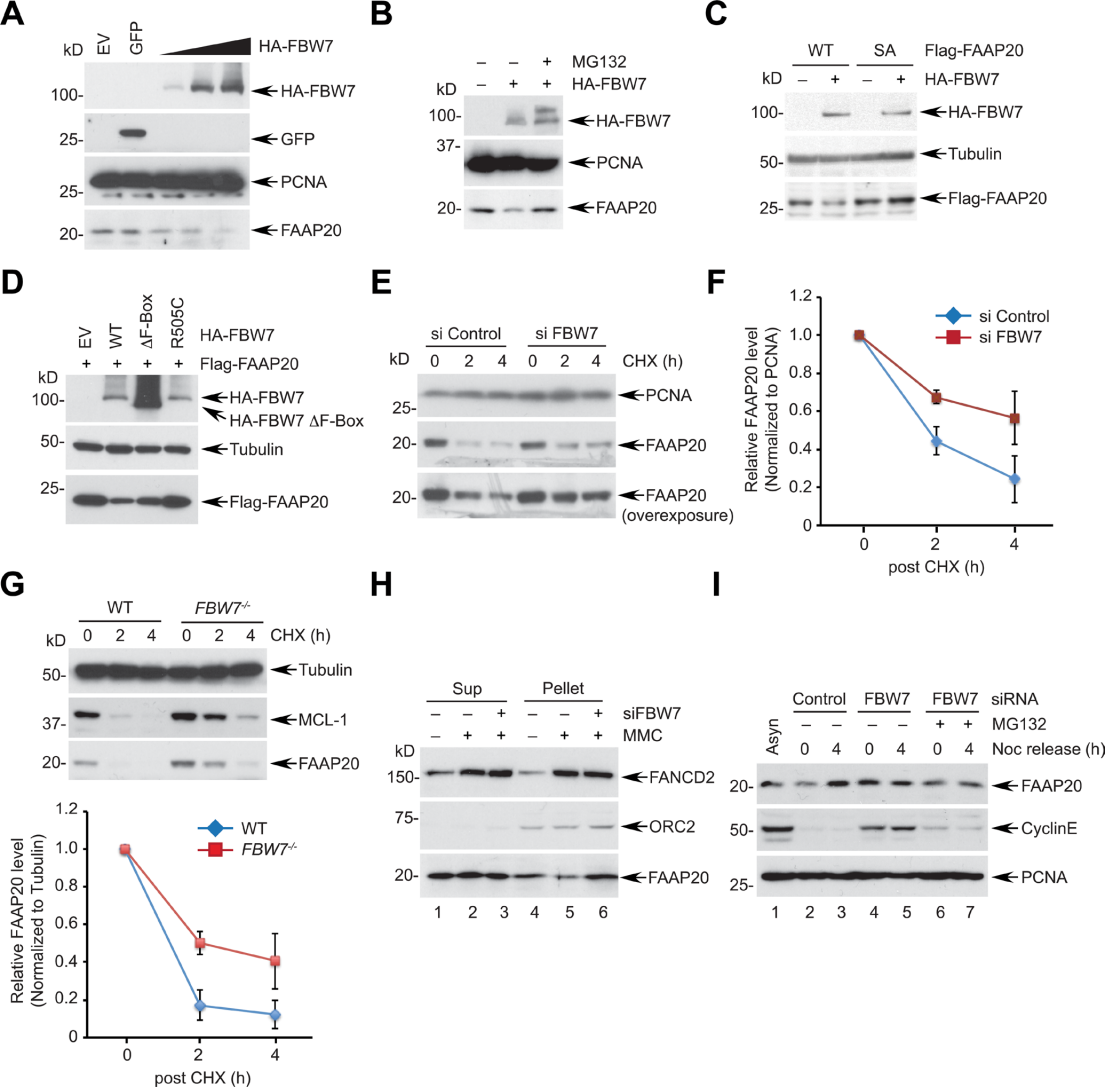
FBW7 is required for proteasomal degradation of FAAP20 **A.** FBW7 overexpression leads to FAAP20 degradation. HeLa cells transfected with increasing levels of the FBW7-encoding plasmid were analyzed by Western blotting. GFP-encoding plasmid served as a negative control. **B.** FAAP20 degradation by FBW7 is proteasome-dependent. HeLa cell lysates transiently expressing HA-tagged FBW7 were analyzed by Western blotting. Where indicated, cells were treated with 10 μM MG132 for 6 h before harvest. **C.** FAAP20 degradation by FBW7 requires an intact CPD motif. HeLa cells transiently expressing Flag-FAAP20 wild-type or SA mutant were overexpressed with FBW7 and cell lysates analyzed by Western blotting. **D.** FBW7 mutants fail to induce FAAP20 degradation. HeLa cells transient expressing Flag-FAAP20 was overexpressed with FBW7 wild-type, ΔF-box (aa 284-324 deletion), or R505C, and cell lysates were analyzed by Western blotting. **E.** Depletion of FBW7 increases the half-life of FAAP20. HeLa cells transfected with siRNA control or *FBW7*-3 for 48 h were treated with 50 μg/mL of cycloheximide for the indicated times and cell lysates analyzed by Western blotting. **F.** Quantification of FAAP20 levels in **E.** by ImageJ. Error bars indicate SD from two independent experiments. **G.** (Top) HCT116 wild-type or *FBW7*^−/−^ cells were treated with 50 μg/mL of cycloheximide for the indicated times and cell lysates analyzed by Western blotting. (Bottom) Quantification of FAAP20 levels by ImageJ. Error bars indicate SD from three independent experiments. **H.** HeLa cells transfected with siRNA control or FBW7 were treated with 100 ng/mL MMC for 24 h and fractionated into cyto/nucleoplasmic (sup) and chromatin-enriched (pellet) fractions using CSK buffer. **I.** HeLa cells transfected with indicated siRNAs were synchronized at the G2/M phase by 100 ng/mL nocodazole and released into G1 following mitotic shake-off. Where indicated, cells were incubated with 10 μM MG132 during release. Cyclin E served as a positive control.

Prompted by the result that the CPD motif is necessary for FAAP20 degradation, we next determined whether FBW7 is responsible for FAAP20 degradation. Overexpression of FBW7, but not GFP control, was sufficient to decrease the endogenous FAAP20 levels in a dose-dependent manner (Figure 2A). An FBW7-dependent decrease of FAAP20 in both HeLa and U2OS cells could be antagonized by proteasome inhibition, suggesting that FAAP20 degradation is mediated by ubiquitin-proteasome signaling (Figure 2B, S2A). The *FAAP20* mRNA levels did not significantly change upon FBW7 overexpression, arguing for posttranscriptional regulation of cellular FAAP20 levels by FBW7 (Figure S2B). By contrast, overexpression of FBW7 failed to decrease the level of the FAAP20 SA mutant, indicating that an intact CPD motif is required for FBW7-dependent FAAP20 degradation (Figure 2C). Furthermore, overexpression of FBW7 F-box deletion or tumor-derived R505C WD40 mutant was not as potent as wild-type in decreasing the FAAP20 levels, suggesting that intact F-box and WD40 domains are required for efficient FAAP20 degradation, which is similar to other known substrates (Figure 2D). Conversely, siRNA-mediated knockdown of FBW7 increased the FAAP20 half-life, as measured by CHX blocking (Figures 2E, 2F, S2C). Moreover, *FBW7* knockout in HCT116 colorectal cancer cells increased the cellular FAAP20 levels and half-life compared with wild-type cells without altering *FAAP20* mRNA levels (Figures 2G, S2D, S2E). Taken together, these results indicate that FBW7 regulates FAAP20 degradation *via* the conserved CPD motif.

Next, we determined if FBW7 regulates FAAP20 levels in the context of the DNA damage response (DDR) and cell cycle. Treatment of an ICL-inducing agent, mitomycin C (MMC), led to the decreased FAAP20 levels specifically in the chromatin-enriched fraction, which was abrogated by FBW7 knockdown (Figure 2H; lane 4 - 6). In addition, the expression level of FAAP20 was lower at the G2/M phase compared with asynchronized cells, which elevated as cells progressed to G1 and S phases (Figure S2F). *FBW7* knockdown increased cellular FAAP20 levels, indicating that FBW7-dependent degradation suppresses FAAP20 levels at the G2/M phase (Figure 2I; compare lane 2 & 4). Together, these results suggest that FAAP20 levels are regulated in DNA damage- and cell cycle-dependent manners by the SCF^FBW7^ complex.

### FBW7 targets FAAP20 for ubiquitination and degradation

**Figure 3:**
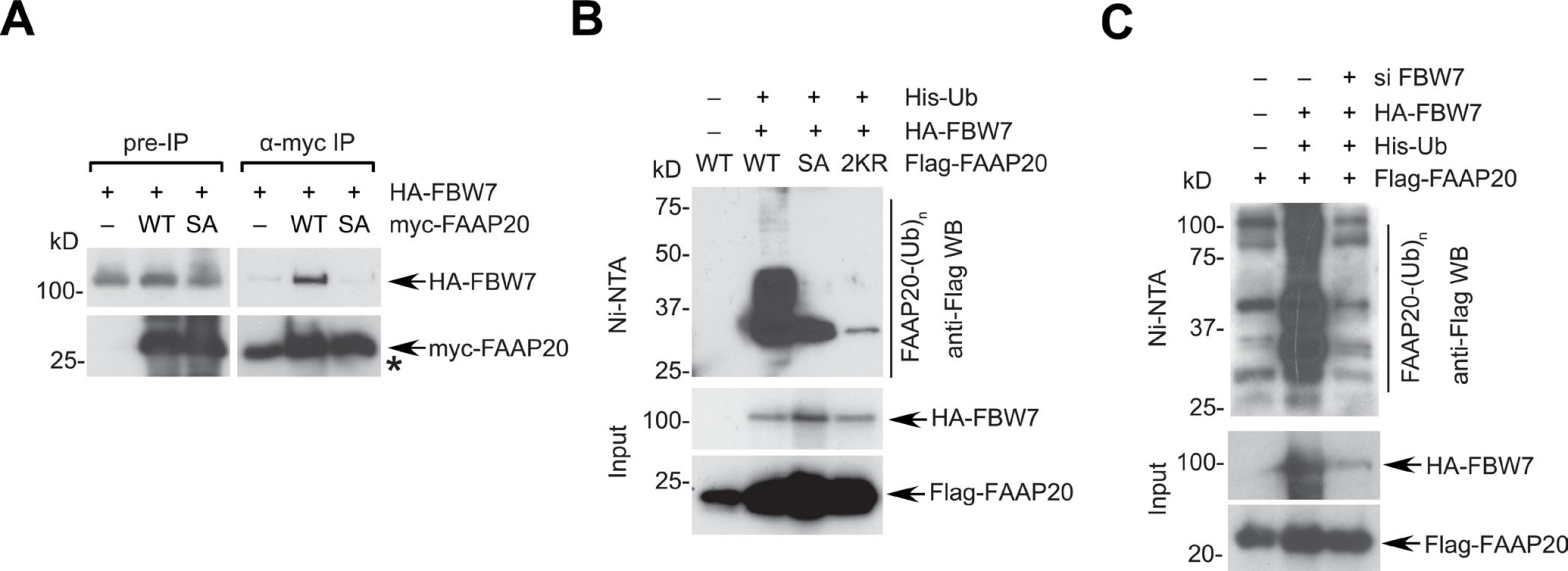
FBW7 induces FAAP20 polyubiquitination and degradation **A.** FBW7 interacts with FAAP20. 293T cells transiently expressing HA-tagged FBW7 and Flag-tagged FAA20 wild-type or SA mutant were treated with 10 μM MG132 for 6 h before harvest and lysed in the presence of phosphatase inhibitor. Cell lysates were subjected to anti-Flag immunoprecipitation and Western blotting. Nonspecific bands co-migrated with myc-FAAP20 signal and were denoted by asterisk. **B.** FBW7 enhances the polyubiquitin conjugates of FAAP20. 293T cells transfected with the indicated plasmids were lysed in denaturing conditions and subjected to Ni-NTA pull-down. Cells were treated with 10 μM MG132 for 4 h before harvest. **C.** The CPD motif of FAAP20 is required for FBW7-dependent FAAP20 polyubiquitination. Ni-NTA pull-down of 293T cell lysates serially transfected with siRNA (control *vs*. FBW7-5) and the indicated plasmids as **B.**.

We next determined whether FBW7 directly functions as a component in the SCF^FBW7^ ubiquitin E3 ligase complex. Interaction of the CPD motif with FBW7 is required for the destruction of substrates. In the presence of proteasome and phosphatase inhibition, myc-tagged FAAP20 was able to co-immunoprecipitate HA-tagged FBW7 whereas the FAAP20 SA mutant failed to do so, suggesting that FBW7 recognizes the phosphorylated CPD motif and interacts with FAAP20 (Figure 3A). The interaction was also observed between *in vitro* transcribed and translated FAAP20 and FBW7 (Figure S3A). An *in vivo* ubiquitination assay that was performed in denaturing condition revealed that exogenous FBW7 is able to enhance the polyubiquitin conjugates of FAAP20 (Figure 3B). By contrast, polyubiquitination was substantially decreased in both FAAP20 SA and K83R/K152 mutants, confirming that the integrity of the CPD motif is essential for FBW7-mediated FAAP20 polyubiquitination (Figure 3B). Moreover, depletion of FBW7 decreased the polyubiquitin conjugates of FAAP20 (Figure 3C). Using GST-tagged ubiquitin binding domain that can capture ubiquitinated substrates from cell extracts [40], we also detected ubiquitinated endogenous FAAP20 induced by FBW7 wild-type, but not the F-Box mutant (Figure S3B). FBW7 could polyubiquitinate FAAP20 *in vitro* as well (Figures S3C, S3D). Together, these data suggest that FBW7 recognizes FAAP20 and promotes its polyubiquitination, which is necessary for proteasomal degradation.

### GSK3β signaling is required for FBW7-mediated FAAP20 degradation

**Figure 4:**
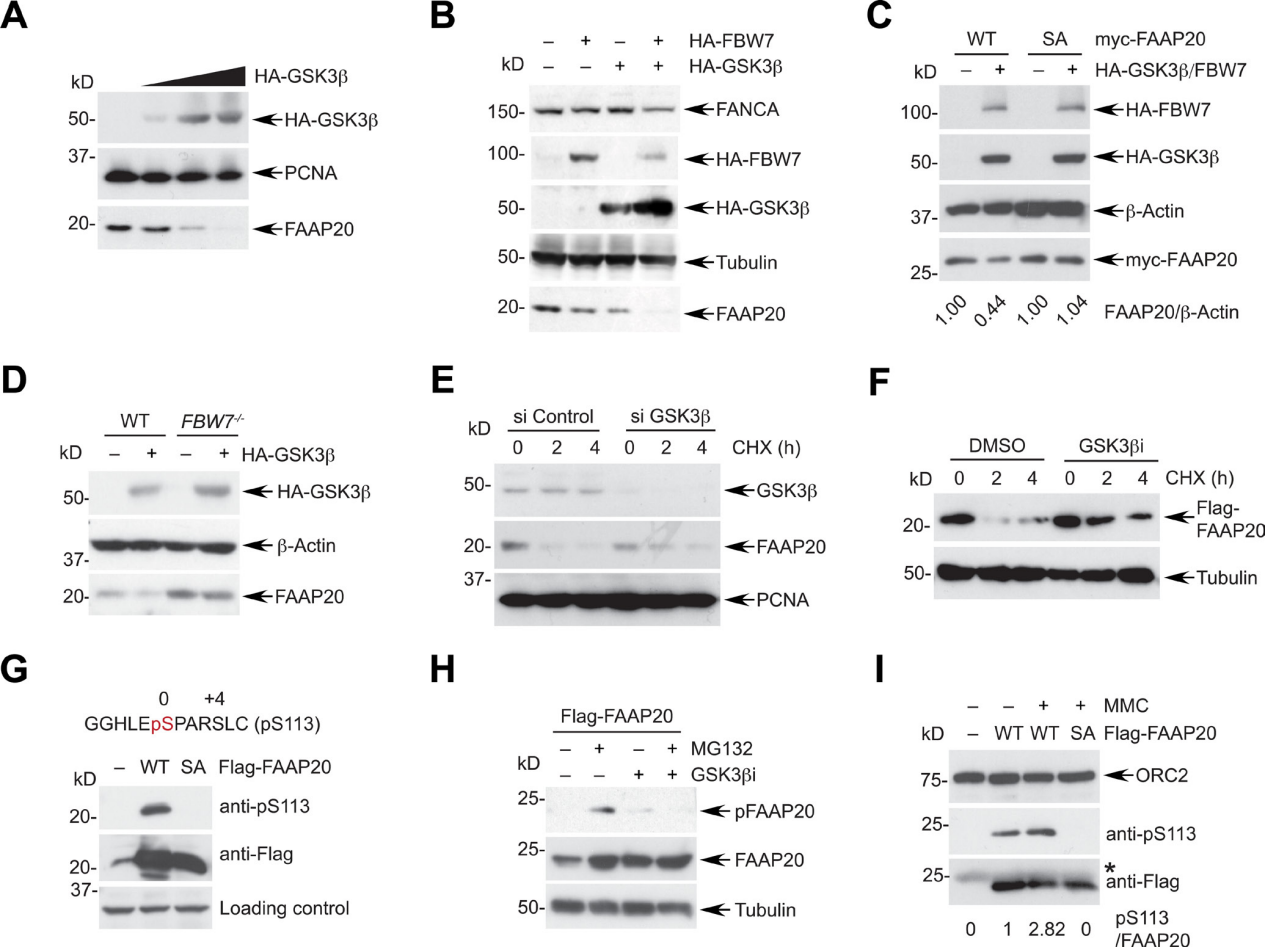
Phosphorylation of FAAP20 by GSK3β mediates FAAP20 degradation **A.** Expression of GSK3β leads to FAAP20 degradation. HeLa cells were transfected with increasing levels of the HA-tagged GSK3β-encoding plasmid, and cell lysates were analyzed by Western blotting. **B.** GSK3β cooperates with FBW7 to induce FAAP20 degradation. HeLa cells were transfected with the indicated plasmids and cell lysates analyzed by Western blotting. **C.** The intact CPD motif of FAAP20 is required for GSK3β to degrade FAAP20. HeLa cells transiently expressing myc-tagged FAAP20 wild-type or SA mutant were transfected with HA-tagged FBW7- and GSK3β-encoding plasmids and cell lysates analyzed by Western blotting. **D.** GSKβ requires downstream FBW7 for FAAP20 degradation. HCT116 wild-type or *FBW7*^−/−^ cells were transfected with the HA-tagged GSK3β-encoding plasmid and cell lysates analyzed by Western blotting. **E.** Depletion of GSK3β increases the half-life of FAAP20. HeLa cells transfected with GSK3β siRNA (*vs*. control) for 48 h were subjected to cycloheximide blocking. **F.** Inhibition of GSK3β activity increases the half-life of exogenous FAAP20. HeLa cells transiently expressing Flag-FAAP20 were pretreated with 20 μM of the GSK3 inhibitor VIII (*vs*. DMSO) for 6 h before treating cycloheximide and analyzed by Western blotting. **G.** Confirmation of the phospho-specific antibody. Cell lysates from 293T cells transiently expressing Flag-FAAP20 wild-type or SA mutant were analyzed by Western blotting. **H.** GSK3β phosphorylates FAAP20 at Ser113. HeLa cells expressing Flag-FAAP20 were treated with 10 μM MG132 and/or 20 μM GSK3 inhibitor VIII for 6 h, and cell lysates were analyzed by Western blotting. **I.** FAAP20 pS113 increases following DNA damage. HeLa cells expressing Flag-FAAP20 variants were left untreated or treated with 100 ng/mL MMC for 16 h, and chromatin-enriched fraction was isolated. The lysates were subjected to anti-Flag immunoprecipitation and analyzed by Western blotting. The ratio of pS113 signal over the total Flag-FAAP20 isolated in the chromatin fraction was indicated.

GSK3β has been shown to phosphorylate the CPD motif of various FBW7 substrates, allowing them to be recognized by FBW7 [16]. Therefore, we determined whether GSK3β signaling regulates FBW7-mediated FAAP20 degradation. As for FBW7 overexpression, the introduction of exogenous GSK3β in HeLa cells decreased the endogenous FAAP20 levels in a dose-dependent manner (Figure 4A). In addition, overexpression of GSK3β, along with FBW7, resulted in a further decrease in the FAAP20 levels compared with the expression of either GSK3β or FBW7 alone, indicating that GSK3β cooperates with FBW7 in controlling FAAP20 proteolysis (Figure 4B). The effect of GSK3β and FBW7 overexpression in inducing FAAP20 degradation was dependent on the intact CPD motif because the FAAP20 SA mutant failed to undergo degradation, whereas the levels of wild-type FAAP20 decreased upon exogenous expression of GSK3β and FBW7 (Figure 4C). Furthermore, GSK3β was able to decrease the cellular FAAP20 levels when overexpressed in wild-type, but not in *FBW7*^−/−^ HCT116 cells, indicating that GSK3β requires downstream FBW7 to regulate the FAAP20 stability (Figure 4D). Conversely, siRNA-mediated knockdown of GSK3β increased the half-life of FAAP20 compared with the control, indicating that GSK3β is necessary for proper degradation of FAAP20 inside cells (Figure 4E). In addition, pharmacological inhibition of GSK3β activity delayed both endogenous and exogenous FAAP20 degradation, indicating that GSK3β-dependent phosphorylation of FAAP20 is required for regulating FAAP20 stability (Figures 4F, S4A).

To further understand the GSK3β signaling that mediates FAAP20 degradation, we generated a phospho-specific antibody that recognizes phosphorylation at serine 113 of FAAP20. The serine in the CPD motif (0 position) is expected to undergo phosphorylation by GSK3β once the +4 position is primed by phosphorylation or through glutamate in the +4 position. The phosphorylation signal at Ser113 was specifically detected in cells expressing wild-type FAAP20, but not in cells with FAAP20 SA mutant as determined by Western blot analysis, confirming the antibody's specificity (Figure 4G). Proteasome inhibition by MG132 treatment increased the FAAP20 levels and phosphorylation of FAAP20 at Ser113. However, treatment with GSK3β inhibitor in the presence of MG132 abrogated the phospho-signal, whereas the FAAP20 levels were elevated, indicating that GSK3β phosphorylates the Ser113 of FAAP20, leading to FAAP20 degradation (Figure 4H). Notably, Ser113 phosphorylation of FAAP20 in the chromatin-enriched fraction increased after MMC treatment, suggesting that turnover of FAAP20 in the functional FA core complex as shown in Figure 2H may be promoted by its phosphorylation during DNA ICL repair (Figure 4I).

GSK3β- and FBW7-dependent substrate degradation is coordinated by upstream signaling. For instance, phosphorylation of GSK3β at Ser9 by the PI3K/AKT mitogen signaling leads to inhibition of GSK3β activity, elevating the levels of FBW7 substrates [41]. Therefore, we evaluated whether FAAP20 degradation is modulated by AKT-dependent GSK3β regulation. Transfection of equal amounts of cDNA encoding GSK3β wild-type or S9A mutant that is refractory to inhibition by AKT signaling revealed that the GSK3β S9A mutant becomes highly stabilized and more potent in degrading FAAP20 when the wild-type has minimal effects (Figure S4B). This data suggest that the potency of GSK3β activity dictates the cellular FAAP20 levels, which may be coordinated by upstream survival signaling. Collectively, these data suggest that phosphorylation of the FAAP20 CPD motif by GSK3β provides a platform for FBW7 to recognize FAAP20 and initiate proteasomal degradation.

### Activity of GSK3β-FBW7 signaling modulates the FA pathway

**Figure 5:**
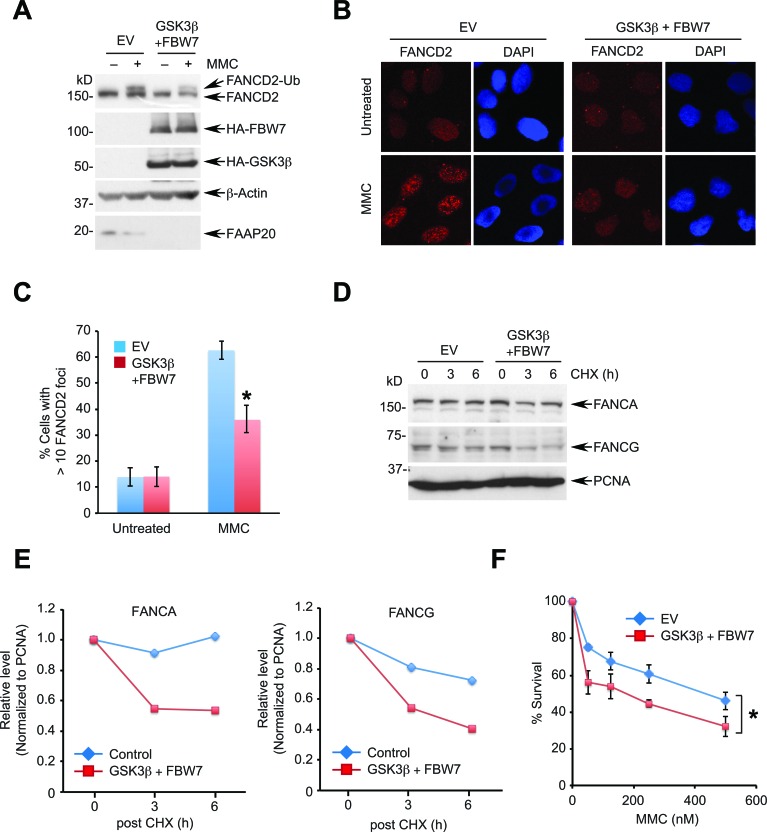
Enhanced GSK3β-FBW7 signaling compromises the FA pathway **A.**, **B.** Overexpression of GSK3β and FBW7 suppresses damage-induced FANCD2 monoubiquitination and foci formation. **A.** HeLa cells coexpressing HA-tagged GSK3β and FBW7 were treated with 1 μM MMC for 8 h and cell lysates analyzed by Western blotting. **B.** U2OS cells coexpressing HA-tagged GSK3β and FBW7 were treated with 100 ng/mL MMC for 16 h and subjected to anti-FANCD2 immunofluorescence. **C.** Quantification of cells in **B.** exhibiting more than 10 FANCD2 foci. Data shown are the mean ± SD from three independent experiments. * *p* < 0.01 compared with vector control. **D.** GSK3β and FBW7 overexpression facilitates the turnover of FANCA and FANCG. HeLa cells expressing HA-tagged GSK3β and FBW7 were treated with 50 μg/mL CHX for the indicated times and analyzed by Western blotting. **E.** Densitometry of FANCA and FANCG levels in **D.** quantitated by ImageJ. **F.** GSK3β and FBW7 overexpression sensitizes cells to a DNA interstrand cross-linking agent. U2OS cells expressing HA-GSK3β and HA-FBW7 were plated to 96 wells, treated with the indicated doses of MMC for 5 days, and cell viability was measured by luminescence assay. Data shown are the mean ± SD from three independent experiments. * *p* < 0.05 compared with control.

FAAP20 directly interacts with the FANCA subunit of the FA core complex and is an essential component of DNA ICL repair *via* the FA pathway [37]. By disrupting the integrity of the FA core complex, deficiency of FAAP20 compromises FANCD2 monoubiquitination, leading to cellular hypersensitivity in response to ICL-inducing agents. Our data demonstrate that GSK3β-FBW7 signaling regulates FAAP20 degradation, suggesting that increased proteolysis of FAAP20 due to the enhanced GSK3β-FBW7 signaling is expected to disrupt the FA pathway. Therefore, we determined the effect of enforced FBW7-dependent proteolytic activity in controlling DNA ICL repair. Coexpression of FBW7 and GSK3β led to substantial reduction in the cellular FAAP20 levels, leading to inhibition of FANCD2 monoubiquitination following MMC treatment (Figure 5A). Monoubiquitinated FANCD2 localizes at the sites of DNA damage and forms damage-inducible nuclear foci, representing activation of the FA pathway [42]. Cells expressing GSK3β and FBW7 showed significantly diminished FANCD2 nuclear foci following DNA damage compared with vector control, suggesting that activation of the FA pathway is compromised (Figures 5B, 5C). These cells exhibited a shorter half-life of FANCA and FANCG, a direct binding partner of FANCA, indicating that loss of FAAP20 by the enhanced GSK3β-FBW7 signaling compromises the integrity of the FA core complex, which leads to a defect in FANCD2 activation (Figures 5D, 5E). Accordingly, cells expressing FBW7 and GSK3β became hypersensitive to MMC (Figure 5F). Collectively, these data suggest that the enhanced GSK3β-FBW7 signaling negatively regulates the FA pathway by decreasing the cellular FAAP20 levels.

### Disruption of FAAP20 homeostasis by the loss of FBW7 causes a defect in the FA pathway

FBW7 promotes degradation of oncoproteins, suppressing their oncogenic potential. Therefore, somatic loss-of-function mutations in *FBW7* are prevalent in a broad range of cancers, which is expected to accelerate tumorigenesis by aberrantly increasing the cellular levels of oncoproteins. As such, it is counterintuitive that FBW7 limits the expression of FAAP20, a core component of DNA repair machinery that is generally considered to function as a tumor suppressor. We reasoned that improper control of FAAP20 due to the loss of FBW7 likely disrupts the homeostasis of FAAP20 and thus the FA core complex required for executing DNA ICL repair. The FA core complex is recruited to sites of DNA damage where it regulates FANCD2 monoubiquitination [43]. However, inactivation of FANCD2 activity, such as by deubiquitination by ubiquitin-specific protease 1 (USP1), is also essential for the stepwise execution of DNA ICL repair [44]. In this respect, failure of timely removal of the FA core complex at the sites of DNA repair may inhibit efficient repair processes, preventing replication fork recovery and a resumption of DNA replication. We thus hypothesized that the dynamics of FANCA during DNA ICL repair are compromised due to the deregulated FAAP20 turnover in the absence of FBW7, leading to a defect in the FA pathway.

**Figure 6:**
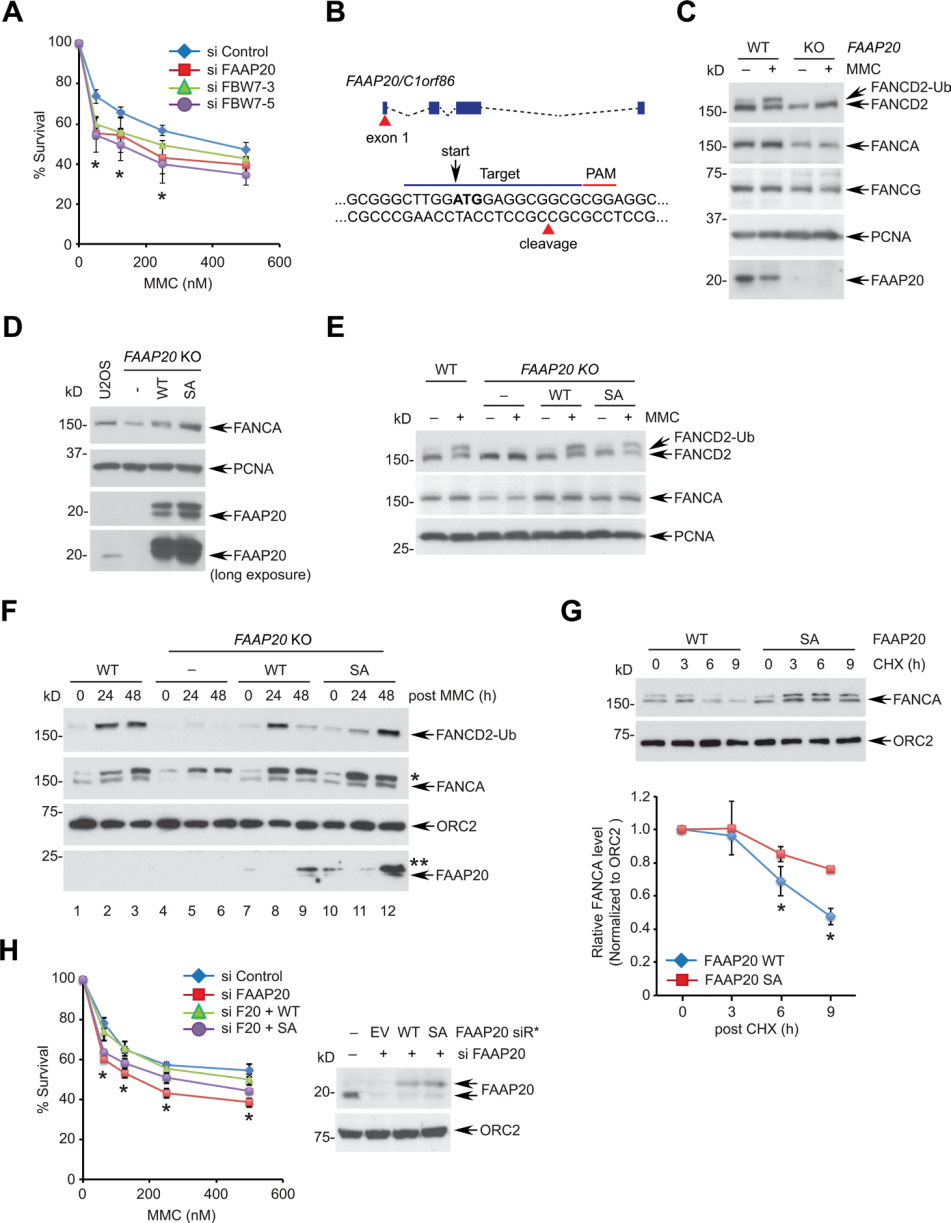
Disruption of the FAAP20 phosphorylation compromises the FA pathway **A.** Depletion of FBW7 hypersensitizes cells to a DNA interstrand cross-linking agent. U2OS cells transfected with indicated siRNA for 48 h were plated to 96 wells, treated with the indicated doses of MMC for 5 days, and cell viability was measured by luminescence assay. FAAP20 depletion served as a positive control. Data shown are the mean ± SD from three independent experiments. * *p* < 0.05 compared with control. **B.** A schematic for the *FAAP20* knockout strategy using CRISPR/Cas9. The 20-nucleotide sgRNA target loci in the exon 1 are marked in blue line along with a PAM sequence in red. The cleavage site for the Cas9 nuclease is shown by red triangle. The ATG start codon is marked in bold with arrow. **C.** U2OS wild-type (vector transfected) or *FAAP20* knockout (KO) clones were treated with 100 ng/mL MMC for 16 h and analyzed by Western blotting. **D.** Western blot analyses of U2OS *FAAP20* KO cells reconstituted with FAAP20 wild-type or SA mutant by retroviral transduction. **E.** Restoration of FANCD2 monoubiquitination by exogenous FAAP20 wild-type or SA mutant. *FAAP20* KO cells stably expressing FAAP20 wild-type or SA mutant were treated with 100 ng/mL MMC for 16 h and analyzed by Western blotting. **F.** Accumulation of FANCA and FANCD2 monoubiquitin in the chromatin-enriched fraction in cells expressing the FAAP20 SA mutant. Indicated U2OS cells were treated with 1 μM MMC for 2 h, replenished with fresh medium to initiate the DNA repair process, and collected at the indicated times. Cells were fractionated, and chromatin-enriched fractions were analyzed by Western blotting. Asterisks denote nonspecific bands. **G.** The half-life of FANCA in the chromatin extends in the cells expressing the FAAP20 SA mutant. (Top) U2OS *FAAP20* KO cells expressing FAAP20 wild-type or SA mutant were treated with 100 ng/mL MMC for 16 h, incubated with 50 μg/mL CHX for the indicated times and fractionated to isolate chromatin-enriched fractions. Cell lysates were analyzed by Western blotting. (Bottom) Quantification of the FANCA level normalized by ORC2. Error bars indicate SD from two independent experiments. * *p* < 0.05 compared with SA. **H.** U2OS cells serially transfected with siRNA and siRNA-resistant FAAP20 variants (siR*) were treated with indicated doses of MMC, and cell viability was measured by luminescence assay. Data shown are the mean ± SD from three independent experiments. * *p* < 0.05 (WT and SA) compared with control except 125 nM for SA (*p* = 0.4940 not significant).

To test this idea, we first determined whether FBW7 is necessary for DNA ICL repair. Depletion of FBW7 using two independent siRNAs led to cellular hypersensitivity to MMC, indicating that FBW7 is required for cellular resistance to ICL-inducing genotoxic stress (Figure 6A). To separate the role of FAAP20 deregulation from the elevated activity of other oncogenic substrates caused by FBW7 loss, we determined the impact of the FAAP20 SA mutant that is refractory to FBW7-dependent degradation on controlling DNA ICL repair. To this end, we knocked out the *FAAP20* gene in U2OS cells by CRISPR/Cas9 genome editing for structure-function analysis (Figure 6B). As expected, the *FAAP20* knockout cells had a decrease in the FANCA and FANCG levels, and they failed to undergo FANCD2 monoubiquitination following MMC treatment, indicating that the function of the FA core complex is impaired (Figure 6C). Reconstitution of knockout cells with wild-type or SA mutant FAAP20 restored the FANCA levels and damage-induced FANCD2 monoubiquitination (Figures 6D, 6E).

We next tested whether defective FAAP20 phosphorylation and degradation causes deregulated FANCA turnover and impairs DNA ICL repair. To this end, we fractionated cells to isolate chromatin-enriched fraction during the course of DNA ICL repair following transient MMC pulse and recovery into fresh medium. FANCA from *FAAP20* KO cells expressing wild-type FAAP20 showed transient accumulation in the chromatin (Figure 6F, lane 7, 8, 9), whereas FANCA with FAAP20 SA mutant exhibited persistent accumulation during the late phase of DNA ICL repair (Figure 6F, lane 10, 11, 12). The FAAP20 SA mutant showed enhanced association in the chromatin compared with the wild-type, implicating that deregulated FAAP20 levels have caused impaired FANCA turnover. Accordingly, monoubiquitinated FANCD2 persisted in the chromatin from cells expressing FAAP20 SA mutant, while it decreased in those expressing wild-type FAAP20 48 h after MMC pulse, indicating that DNA ICL repair is delayed. FANCA in the chromatin-enriched fraction from cells expressing the FAAP20 SA mutant exhibited a prolonged half-life in comparison to wild-type FAAP20-expressing cells following MMC treatment, indicating that the turnover of FANCA during DNA repair is delayed due to defective FAAP20 degradation (Figure 6G). Accordingly, cells expressing the FAAP20 SA mutant could not fully complement the hypersensitivity of FAAP20-depleted cells to MMC, albeit better than vector-expressing cells, indicating that regulated turnover of FAAP20 is at least one of the important components of DNA ICL repair (Figure 6H).

Taken together, these results indicate that disruption of FAAP20 homeostasis by either FBW7 deficiency or a FAAP20 phosphorylation defect leads to compromised DNA ICL repair by prolonged FANCA accumulation at the site of DNA damage. In this sense, FAAP20 degradation, which is initiated by its phosphorylation at the CPD motif following DNA damage may be a key regulatory signal for removing FANCA from chromatin in a timely fashion in order to complete DNA ICL repair.

## DISCUSSION

In this study, we present evidence that the FA pathway is regulated by SCF^FBW7^ ubiquitin E3 ligase-mediated proteolysis. We identified a conserved phospho-degron motif, called the CPD motif, in FAAP20 and demonstrated that FAAP20 is a functional target of SCF^FBW7^. The serine 113 of the CPD motif is phosphorylated by GSK3β, which in turn is recognized by the F-box protein FBW7, leading to proteasomal degradation. Loss of the CPD phosphorylation or mutation in the WD40 domain of FBW7 abolishes GSK3β- and FBW7-dependent FAAP20 destruction, indicating that the GSK3β-FBW7 proteolytic signaling axis regulates FAAP20 turnover. Overexpression of GSK3β and FBW7 was sufficient to destabilize FAAP20, impair FANCD2 activation mediated by the FA core complex, and disrupt DNA ICL repair, supporting the idea that SCF^FBW7^-dependent proteolysis directly regulates the FA pathway via regulating FAAP20 degradation.

### Regulation of the FANCA-FAAP20 interaction dynamics during DNA ICL repair

**Figure 7:**
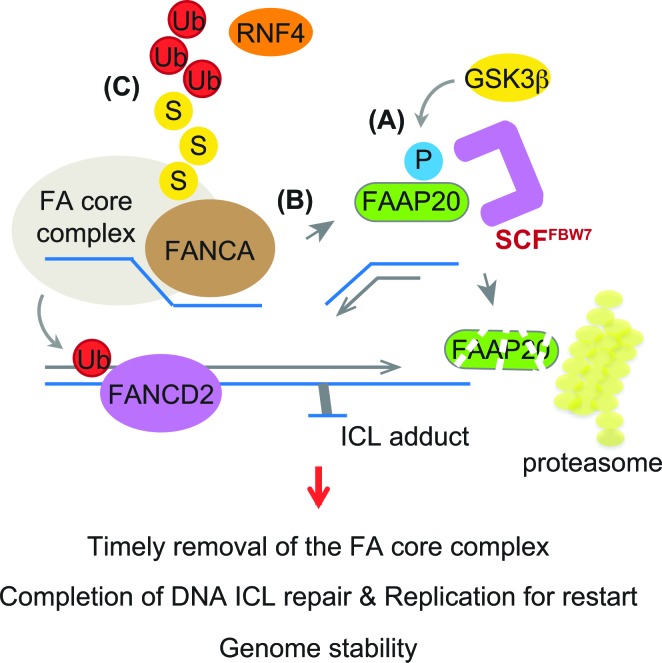
Model depicting the role of FBW7 in regulating the FA pathway The FA core complex needs to be removed from the sites of repair in a timely fashion to complete DNA ICL repair. **A.** GSK3β-dependent phosphorylation leads to FAAP20 degradation mediated by SCF^FBW7^ and the proteasome. **B.** This allows for FAAP20 dissociation from the FANCA subunit of the FA core complex. **C.** FANCA in the absence of FAAP20 leads to SUMO-dependent degradation mediated by STUbL RNF4, destabilizing the FA core complex. Loss of FBW7 function or a defect in FAAP20 phosphorylation prevents this process, causing disruption of the FA pathway and genome instability.

Our study reveals a new regulatory feature of the FA pathway that is controlled by FBW7-dependent proteolysis, namely phosphorylation-dependent regulation of the FA core complex for completing DNA ICL repair. We propose that FANCA turnover, which is prompted by FAAP20 phosphorylation and degradation, is required for inactivation of the FA core complex and its clearance from the sites of DNA repair (Figure 7). We have previously shown that the loss of FAAP20 interaction with FANCA leads to exposure of the FANCA degradation motif, resulting in FANCA SUMOylation and subsequent degradation [39]. RNF4, a SUMO-targeted ubiquitin E3 ligase (STUbL), is responsible for recognizing SUMO modification of FANCA and conjugating ubiquitin chains for proteasomal degradation. Accordingly, depletion of RNF4, which deregulates FANCA turnover, disrupts the FA pathway. Our results indicate that temporal regulation of FAAP20 phosphorylation at the CPD motif during DNA repair is a key regulatory step for controlling the FANCA-FAAP20 interaction dynamics. Aberrant accumulation of FANCA at the sites of DNA repair could prevent completion of the repair process and recovery of the replication forks, leading to replication fork collapse and genome instability. Consistent with this idea, we demonstrated that cells expressing non-phosphorylatable FAAP20 mutant accumulate FANCA in the chromatin during the late phase of DNA ICL repair, leading to the disruption of the FA pathway. Deregulation of FAAP20 phosphorylation may impact FANCD2 ubiquitination directly by disrupting the function of the FA core complex. Several regulatory mechanisms have been proposed to complete the FA pathway by inactivation of the FA factors. USP1-UAF1, a deubiquitinating enzyme complex, removes monoubiquitin from FANCD2 to inactivate it [45, 46]. FANCM, a docking module of the FA core complex to DNA, is degraded, which results in release of the FA core complex in a cell cycle-dependent manner [47]. Regulation of the FANCA-FAAP20 interaction dynamics by FAAP20 degradation via GSK3β-FBW7 signaling is another important layer of regulation that is necessary for the timely completion of DNA ICL repair.

Regulated proteolysis of DNA repair factors to control distinct DNA repair steps has been reported in other DNA repair mechanisms as well. SUMOylation and subsequent degradation of MDC1 by RNF4 is required for timely disassembly of DNA damage factors and HR during the DNA double-strand break (DSB) repair [48]. RNF4 also regulates turnover and exchange of SUMOylated RPA, which promotes RAD51 loading to single-stranded DNA, a prerequisite step for HR [49, 50]. Furthermore, activated FANCI-FANCD2 (ID) complex is removed from DNA lesions by RNF4 during DNA repair, which helps maintain effective dose of the activated ID complex required for the repair process [51]. Conjugation of the ubiquitin-like protein NEDD8 has been shown to promote Ku ubiquitination and release from damage sites following DNA repair, which is required for non-homologous end joining after DSB induction [52]. These examples emphasize the notion that maintaining balanced levels of DNA repair factors is essential for the proper execution of the DNA repair processes, and proteolysis modulated by integrated posttranslational modifications plays a key role in regulating these events. Impeding proper turnover of DNA repair factors at the sites of repair may result in persistent DNA damage signaling or hyperactivation of nuclease function. The spatiotemporal regulation of DNA repair factors by proteolysis may also provide selectivity in the total cellular pool of proteins, eliminating only a functional population of DNA repair factors that actively participate in the DNA repair processes.

### Regulation of GSK3β-FBW7 signaling

One of the unique features of FBW7-dependent proteolysis is that the degradation of substrates is regulated by upstream phosphorylation, which creates an optimal docking site for FBW7 recognition. We showed that phosphorylation of Ser113 by GSK3β is enhanced following genotoxic stress and is essential for FAAP20 degradation. Interestingly, the activity of GSK3β is inhibited by Ser9 phosphorylation through the PI3K/AKT mitogen-signaling pathway, suggesting that the FA pathway might be coordinated by cell growth and proliferation, especially during malignant transformation that is in turn governed by oncogene-induced replication stress. Coordination of FBW7 with the DDR has been reported. To restrict cellular proliferation during genotoxic challenge, FBW7-mediated *c*-Myc degradation is enhanced following UV irradiation through dissociation of USP28 from FBW7, a DUB that is associated with SCF^FBW7^ and prevents *c*-Myc from destruction [53, 54]. Nevertheless, the mechanism by which GSK3β-FBW7 signaling is regulated in the context of the DDR remains largely unexplored. Of note, GSK3β and FBW7 contain S/TQ motifs that are phosphorylated by the ATM/ATR kinase, and some of these have been validated by large-scale proteomic analysis [55], indicating that DNA damage signaling may directly activate selective protein degradation pathways that are required for cell cycle checkpoints and DNA repair. FBW7 is also a direct transcriptional target of p53 [56], raising the possibility that the activity of FBW7 may be induced by the DDR coordinated by p53, allowing for control of both the cell cycle and DNA repair.

### The role of FBW7-dependent proteolysis in DNA repair and genome stability

Most FBW7 substrates are proto-oncogenes that are implicated in cancer development. It is interesting to note that FBW7, a tumor suppressor, promotes degradation of a DNA repair protein that is known to guard genome integrity. As revealed in our study, loss of FBW7 leads to disruption of cellular homeostasis of FAAP20, causing a functional defect in the FA pathway. In this case, increased levels of tumor suppressors due to impaired protein turnover could be as detrimental as elevated oncoproteins caused the loss of FBW7 activity. Somatic loss-of-function mutations in *FBW7* are prevalent in multiple cancers, which is expected to accelerate tumorigenesis by aberrantly increasing the cellular levels of oncoproteins. In addition to hyper-proliferation due to elevated levels of *c*-Myc and Cyclin E, loss of FBW7 function may promote tumorigenesis by increasing genome instability caused by direct modulation of DNA repair proteins. Furthermore, given that loss of FBW7 activity is associated with acquired chemoresistance [57], altered DNA repair activity in FBW7-deficient cancers may contribute to increased tolerance to the toxicity of DNA-damaging chemotherapeutic agents during chemotherapy. p53 has been shown to suppress the genome instability caused by the loss of FBW7 [58]. Deregulated DNA repair activity may cooperate with p53 loss to overcome the anti-cancer barrier induced by the DDR. Therefore, our study provides insights into how loss of FBW7 is associated with genome instability by establishing a new connection between FBW7 and the FA pathway. FBW7 could have multiple substrates involved in various DNA repair processes. For instance, it was recently shown that bloom helicase (BLM) is targeted by FBW7 during mitosis [59]. As FBW7 deficiency exhibits more profound cellular hypersensitivity to MMC compared with cells with the FAAP20 SA mutant, we do not exclude the possibility that FBW7 may have other substrates required for DNA ICL repair. Comprehensive understanding of the role of FBW7 in regulating DNA repair will ultimately allow us to develop therapeutic strategies that exploit aberrant regulation of DNA repair in cancer cells that are caused by the loss of FBW7, as well as to sensitize cancer cells that are resistant to chemotherapy by restoring FBW7 activity.

## MATERIALS AND METHODS

### Cell culture and plasmid construction

HeLa, U2OS, and 293T cells were acquired from American Type Culture Collection (ATCC) and cultured in Dulbecco's Modified Eagles Medium (DMEM) supplemented with 10 % fetal bovine serum (FBS) and 1 % Penicillin-Streptomycin following standard culture conditions and procedures. HCT116 colorectal cancer cell lines, generous gifts from Dr. Vincent Yang (Stony Brook Medicine, NY), were cultured in McCoy's 5A medium with 10 % FBS. FAAP20 constructs were as previously described [37]. Human *FBW7* and *GSK3β* cDNA were acquired from the Dana-Farber/Harvard Cancer Center DNA Resource Core (HsCD00399882) and Open Biosystems (MHS6278-202828006), respectively. The full-length or deleted cDNA was PCR-amplified and subcloned into modified pcDNA3-Flag, pcDNA3-HA (Invitrogen), or pMSCV-Flag-HA (Clontech) vectors. Point mutations were introduced using the QuikChange II XL Site-Directed Mutagenesis Kit (Agilent Technologies). All the constructs were verified by DNA sequencing.

### DNA and siRNA transfection

Plasmid transfection was performed using GeneJuice (Millipore) according to the manufacturer's protocols. siRNA duplexes were synthesized by Qiagen, except FBW7-5, which was obtained from Ambion, and GSK3β, which was obtained from Cell Signaling; they were transfected at 25 nM using Lipofectamine RNAiMAX (Invitrogen). The siRNA target sequences used in this study are as follows: Control 5′-GGGTATCGACGATTACAAATT-3′; FAAP20 5′-CACGGTGAGCCCGGAGCTGAT-3′; FBW7-3 5′-GTGGAATGCAGAGACTGGAGA-3′; and FBW7-5 5′-CGGGTGAATTTATTCGAAATT-3′. GSK3β sequence information is not available. The siRNA-resistant FAAP20 construct (in pMSCV) was generated by introducing seven silent mutations (shown as lower case in 5′-gACtGTtAGtCCtGAaCTaAT-3′).

### Chemicals

Mitomycin C, Z-Leu-Leu-Leu-al (MG132), cycloheximide, thymidine, and nocodazole were purchased from Sigma-Aldrich. The GSK3β inhibitor VIII was purchased from EMD Millipore. The phosphatase inhibitor cocktail was obtained from Cell Signaling. Drugs were used at the concentrations indicated in the figure legends.

### Western blotting and antibodies

Cells were lysed with NETN300 buffer (1 % NP40, 300 mM NaCl, 0.1 mM EDTA, and 50 mM Tris [pH 7.5]) supplemented with protease inhibitor cocktail (Roche), resolved by SDS-PAGE gels and transferred onto PVDF membranes (Millipore), and then antibodies were detected using the enhanced chemiluminescence method (Western Lightening, Perkin Elmer). The following antibodies were used for Western blot analysis: FAAP20 (Sigma-Aldrich), FANCD2 (FI-17, Santa Cruz), PCNA (PC-10, Santa Cruz), Cyclin E (H-12, Santa Cruz), FANCA (Bethyl), FBW7 (Bethyl), FANCG [33], MCL-1 (Cell Signaling), GSK3β (Cell Signaling), HA (6E2, Cell Signaling), ubiquitin (P4D1, Cell Signaling), CUL1 (Cell Signaling), Flag (M2, Sigma-Aldrich), c-Myc (9E10, Sigma-Aldrich), β-Actin (Cell Signaling), Tubulin (Sigma-Aldrich), and ORC2 (BD Pharmingen). pS113 FAAP20 phospho-specific antibody was generated by Genscript using the GGHLEpSPARSLC peptide to immunize rabbits.

### Subcellular fractionation

Subcellular fractionation was performed as previously described [37]. Briefly, cells were incubated with 0.1 % Triton X-100 cytoskeleton (CSK) buffer (10 mM Tris [pH 6.8], 100 mM NaCl, 300 mM sucrose, MgCl_2_, 1 mM EGTA, 1 mM EDTA, and 0.1% Triton X-100) for 5 min on ice. After centrifugation at 1,500 g for 5 min, supernatant was separated from the pellet enriched with chromatin. Pellets were resuspended with PBS and resolved in equal amount of 2X boiling lysis buffer (50 mM Tris-Cl [pH 6.8], 2 % SDS, and 850 mM β-mercaptoethanol), which was followed by 10 min of boiling.

### Co-immunoprecipitation

293T cells that were treated with 10 μM MG132 for 6 h were lysed with NETN150 buffer (1 % NP40, 150 mM NaCl, 0.1 mM EDTA, 50 mM Tris [pH 7.5]) in the presence of phosphatase inhibitor cocktail (Cell Signaling) and protease inhibitor, and they were centrifuged at 15,000 rpm for 10 min at 4°C. Lysates (4 %) were saved as pre-IP. Cell lysates were incubated with anti-Flag affinity gel (M2, Sigma-Aldrich) for 4 h followed by five washes with NETN150 buffer. Resins were boiled in 2X Laemmli sample buffer and subjected to SDS-PAGE. For co-immunoprecipitation of *in vitro* transcribed and translated proteins, A total of 250 ng of pcDNA3-Flag-FAAP20 and pcDNA3-HA-FBW7 plasmids were incubated with 10 μL of TnT^®^ T7 Quick Coupled Transcription/Translation Master Mix (Promega) at 30°C for 70 min to produce proteins.

### RT-qPCR

RNA was isolated using TRIzol (Invitrogen). cDNA synthesis was performed using a high-capacity cDNA reverse transcription kit (Applied Biosystems) according to the manufacturer's protocols. The mRNA levels were quantified using a Fast SYBR Green Master Mix (Applied Biosystems) and StepOnePlus Real-time PCR system (Applied Biosystems).

### Fluorescence microscopy

For FANCD2 foci visualization, U2OS cells grown in chamber slides (BD Falcon) were pre-extracted with PBS/ 0.3 % Triton X-100 and fixed with 4 % paraformaldehyde in PBS. Following three washes with PBS, cells were blocked with 2 % bovine serum albumin (BSA, Sigma-Aldrich). Cells were incubated with an anti-FANCD2 primary antibody (1: 500, Novus Biologicals) in PBS/ 1 % BSA for 2 h at RT, washede three times in PBS, and incubated with 1:1000 Alexa Fluor^®^ 568 goat anti-mouse IgG secondary antibody (Molecular Probes) for 1 h at RT. Cells were mounted with DAPI-containing mounting medium (Vector Lab), and images were captured using a Zeiss LSM 510 inverted confocal microscope with Zeiss LSM software. Images were processed using Adobe Photoshop CS6.

### Ubiquitin assay

*In vivo* ubiquitin assays were performed in denaturing conditions. 293T cells that were treated with 10 μM MG132 for 4 h were resuspended with PBS/1 % SDS, snap-frozen in liquid nitrogen, and boiled for 15 min. Cell lysates were diluted 10 times with PBS and centrifuged at 15,000 rpm for 15 min at 4°C. Lysates (4 %) were saved for input, and lysates were incubated with HisPur^TM^ Ni-NTA Resin (Thermo Fisher) in the presence of 10 mM imidazole (Sigma-Aldrich) for 3 h at 4°C, followed by five washes with PBS/0.1 % SDS, 10 mM imidazole. Resins were boiled in 2X Laemmli sample buffer and subjected to SDS-PAGE. To detect *in vitro* ubiquitination of FAAP20, Flag-HA-FBW7 was stably expressed in 293T cells and purified using anti-Flag M2 beads, and the SCF^FBW7^ E3 complex was eluted by 500 μg/mL Flag peptide (Sigma). The reactions were performed with 50 ng E1 (UBE1), 200 ng E2 (UbcH5c), purified E3, 5 μg ubiquitin, 2 mM ATP, 5 mM MgCl_2_, and 2 mM DTT along with *in vitro* translated myc-FAAP20 in a final volume of 30 μL at 30°C for 2 h with continuous agitation. The reaction was terminated by PBS/1 % SDS, boiled for 10 min, and diluted 10 times for anti-myc IP. To detect ubiquitination of endogenous FAAP20, MG132-treated cells were lysed in NETN150 buffer in the presence of 5 μg of GST-TUBE2 (Tandem Ubiquitin Binding Entities 2; Lifesensors), and cell lysates were incubated with glutathione Sepharose 4B (GE Healthcare) at 4°C for 3 h. Washed resins were boiled in 2X Laemmli SDS sample buffer for SDS-PAGE.

### Drug sensitivity assay

Cells in 6-well plates were transfected with siRNA oligos and seeded on 96-well plates next day. Cells were treated with MMC in duplicates at 48 h after transfection, and cell viability was determined using the CellTiter-Glo luminescent cell viability assay (Promega) 5 days after continuous drug treatment. Luminescence was measured using a Centro LB960 Microplate Luminometer (Berthold Technologies) and Mikrowin 2000 software.

### Generation of *FAAP20* knockout cells

A pair of oligos containing the *FAAP20* sgRNA targeting sequence was cloned into pSpCas9(BB)-2A-Puro (pX459; a gift from Feng Zhang, Addgene plasmid # 48139) and transfected into U2OS cells using GeneJuice (Millipore) [60]. Control cells were transfected with an empty vector. Subsequently, cells were selected with 2 μg/mL puromycin and replated into 96 wells after recovery for clonal selection. Selected clones were analyzed by anti-FAAP20 Western blot analysis to confirm successful knockout. For generating stable cell lines reconstituted with wild-type or SA mutant, *FAAP20* knockout cells were retrovirally transduced with pMSCV-FAAP20 constructs and selected with 2 μg/mL puromycin. Viruses were collected from 293T cells that were co-transfected with pMSCV-FAAP20, pCMV-Gag/Pol and pCMV-VSV-G and then infected with 8 μg/mL polybrene (Sigma-Aldrich).

### Statistical analysis

*P* values for statistical analyses were obtained using Student's *t* test.

## SUPPLEMENTARY MATERIAL FIGURES


