# Supplementary material for: FBW7 regulates DNA interstrand cross-link repair by modulating FAAP20 degradation
Source: Oncotarget. 2016 May 25;7(24):35724–40. doi: 10.18632/oncotarget.9595 (PMC5094957; doi:10.18632/oncotarget.9595)
Supplement: Supplementary file 1 [file oncotarget-07-35724-s001.pdf]

FBW7 regulates DNA interstrand cross-link repair by modulating FAAP20 degradation

Supplementary Material

A

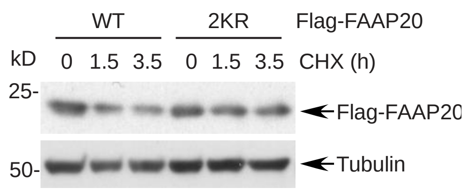

Supplementary figure S1 – related to Figure 1

(A) Mutation of lysine residues in FAAP20 prevents FAAP20 degradation. Flag-tagged wild-type (WT) or K83R/K152R (2KR) mutant were transiently expressed in HeLa cells, treated with 50 μg/mL of cycloheximide (CHX) for the indicated times, and cell lysates were analyzed by Western blotting.

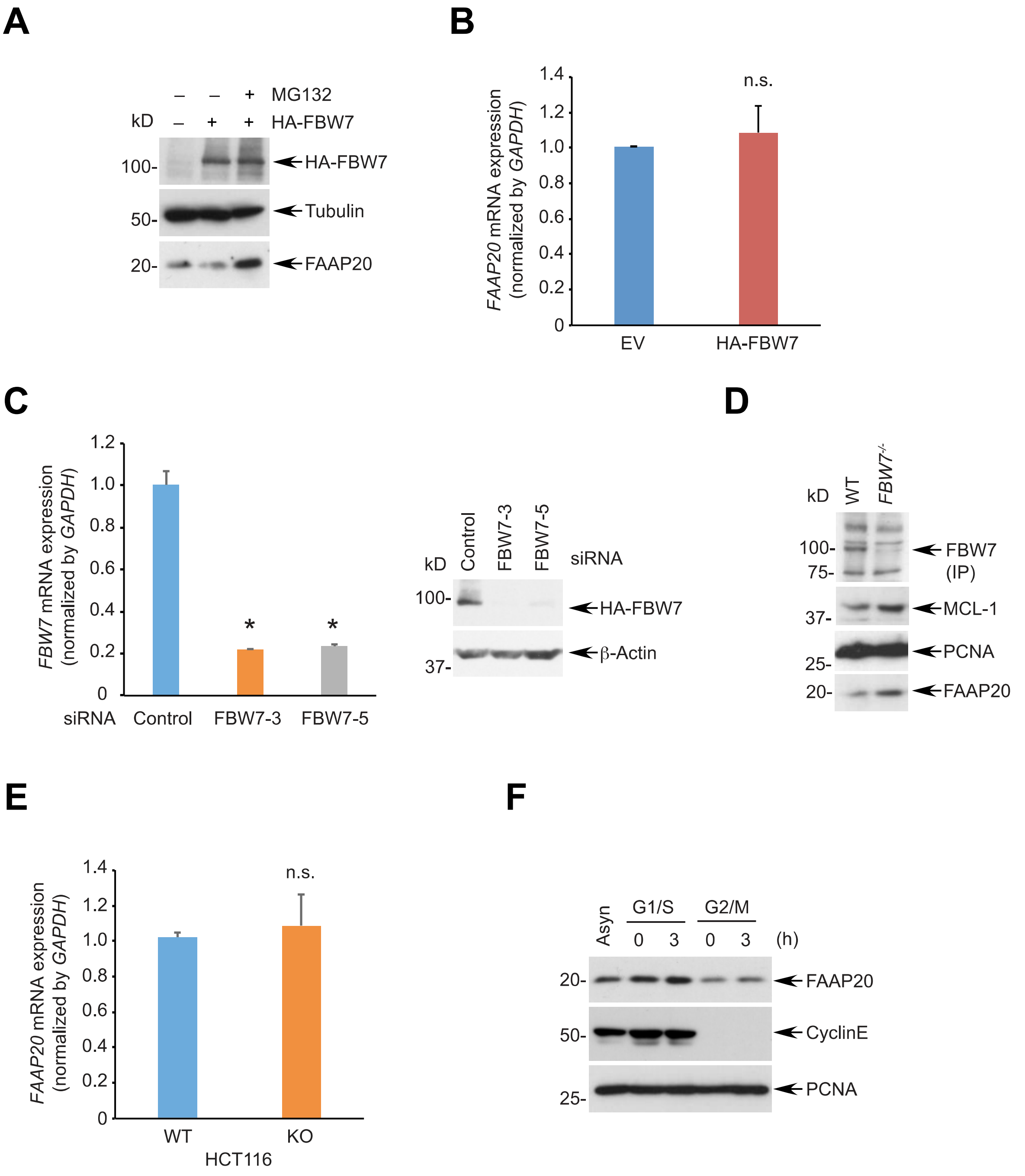

Supplementary figure S2 – related to Figure 2

(A) FAAP20 degradation by FBW7 is proteasome-dependent. U2OS cell lysates transiently expressing FBW7 was analyzed by Western blotting. Where indicated, 20 μM MG132 was treated for 6 h before harvest. **(B)** Decreased FAAP20 levels by FBW7 overexpression are posttranscriptional. *FAAP20* mRNA expression was analyzed by qPCR from U2OS cells transfected with empty vector (EV) or HA-FBW7-encoding plasmid for 48 h. *FAAP20* mRNA levels were normalized by *GAPDH* mRNA. Data shown are mean ± SD from three independent experiments. n.s.: not significant. **(C)** Knockdown efficiency of *FBW7* siRNA. (left) HeLa cells were transfected with siRNA control or two independent *FBW7* siRNA for 48 h, and mRNA expression was analyzed by qPCR. *FAAP20* mRNA levels were normalized by *GAPDH* mRNA. Data shown are mean ± SD from three independent experiments. * $p < 0.01$ compared to scramble control. (right) Western blot analysis of HeLa cells expressing HA-FBW7 transfected with *FBW7* siRNA. Depletion of exogenous HA-FBW7 was confirmed by anti-HA Western blotting. **(D)** Western blotting analysis of HCT116 wild-type and FBW7$^{-/-}$ cells. For FBW7, FBW7 was immunoprecipitated with 2 μg of anti-FBW7 antibody and immunoblotted with the same antibody. **(E)** *FAAP20* mRNA expression was analyzed by qPCR from HCT116 cells. Data shown are mean ± SD from three independent experiments. n.s.: not significant. **(F)** Cell cycle dependent-change of FAAP20 levels. HeLa cells were either synchronized at the G1/S boundary by 2 mM double thymidine block or at the G2/M phase by 100 ng/mL nocodazole. Cells were released into fresh medium to allow progress to S and G1 phases, respectively, and analyzed by Western blotting.

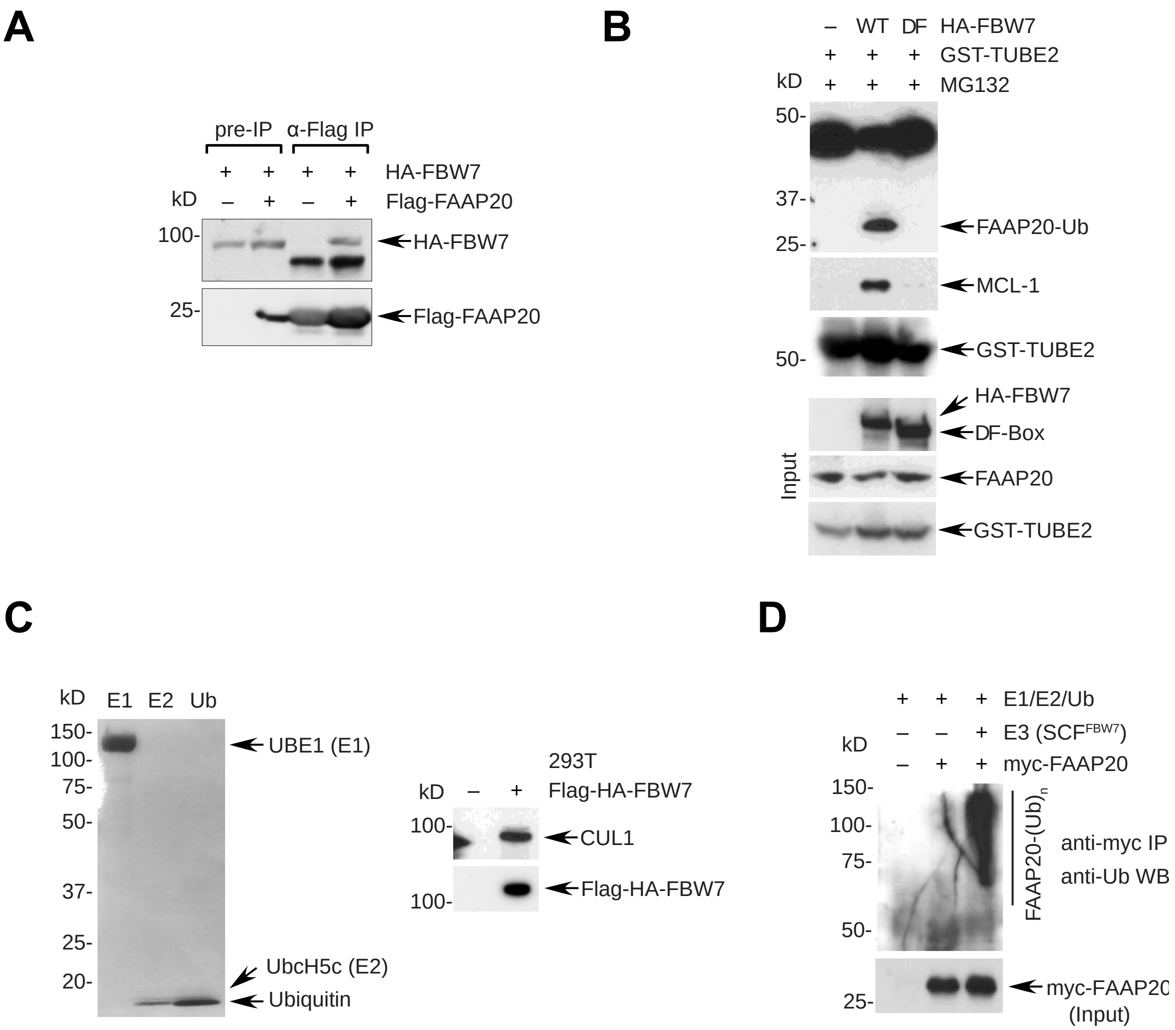

Supplementary figure S3 – related to Figure 3

(A) FBW7 interacts with FAAP20 *in vitro*. HA-tagged FBW7 and Flag-FAAP20 were *in vitro* transcribed and translated using reticulocyte lysates, and subjected to anti-Flag co-immunoprecipitation. **(B)** Endogenous FAAP20 is ubiquitinated. 293T cells were transfected

with HA-FBW7 wild-type or F-Box mutant, treated with 10 μM MG132 for 6 h, and cell lysates were incubated with 5 μg of GST-TUBE2 (Tandem Ubiquitin Binding Entities 2) and glutathione affinity resin. **(C)** Reagents used in *in vitro* ubiquitin assay. Left) Coomassie blue staining of E1, E2, and ubiquitin. Right) 293T cells stably expressing Flag-HA-FBW7 were subjected to anti-Flag immunoprecipitation, and immune complex was eluted by 500 μg/mL Flag peptide. The interaction of exogenous FBW7 with endogenous CUL1 is shown. **(D)** FAAP20 is uniquitinated *in vitro*. *In vitro* transcribed and translated myc-tagged FAAP20 was incubated with E1 (50 ng), E2 (200 ng), ubiquitin (5 μg), and the purified E3 complex in the presence of 2 mM ATP at 30 °C. The reaction sample was denatured with SDS and myc-FAAP20 was immunoprecipitated with anti-myc IP and ubiquitinated FAAP20 was analyzed by anti-ubiquitin Western blotting.

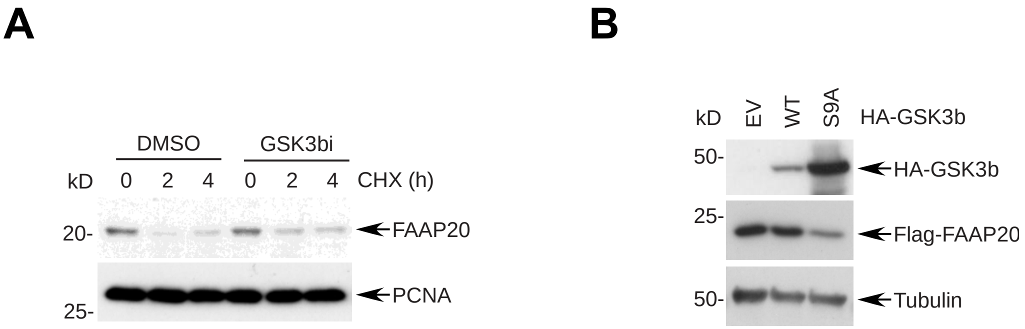

Supplementary figure S4 – related to Figure 4

(A) Inhibition of GSK3β activity increases the half-life of endogenous FAAP20. HeLa cells were pretreated with 20 μM of the GSK3 inhibitor VIII (vs. DMSO) for 6 h before treating cycloheximide and analyzed by Western blotting. **(B)** GSK3β activity regulates FAAP20 degradation. HeLa cells were transfected with GSK3β wild-type or S9A non-phosphorylatable mutant-encoding plasmid, and cell lysates were analyzed by Western blotting.
